# Supplementary material for: Arachidonic acid-regulated calcium signaling in T cells from patients with rheumatoid arthritis promotes synovial inflammation
Source: Nat Commun. 2021 Feb 10;12:907. doi: 10.1038/s41467-021-21242-z (PMC7875984; doi:10.1038/s41467-021-21242-z)
Supplement: Supplementary file 1 — Supplementary Information [file 41467_2021_21242_MOESM1_ESM.pdf]

Supplementary information

Arachidonic acid-regulated calcium signaling in T cells from patients with rheumatoid arthritis promotes synovial inflammation

Zhongde Ye, Yi Shen, Ke Jin, Jingtao Qiu, Bin Hu, Rohit R. Jadhav, Khushboo Sheth, Cornelia M. Weyand, Jörg J. Goronzy

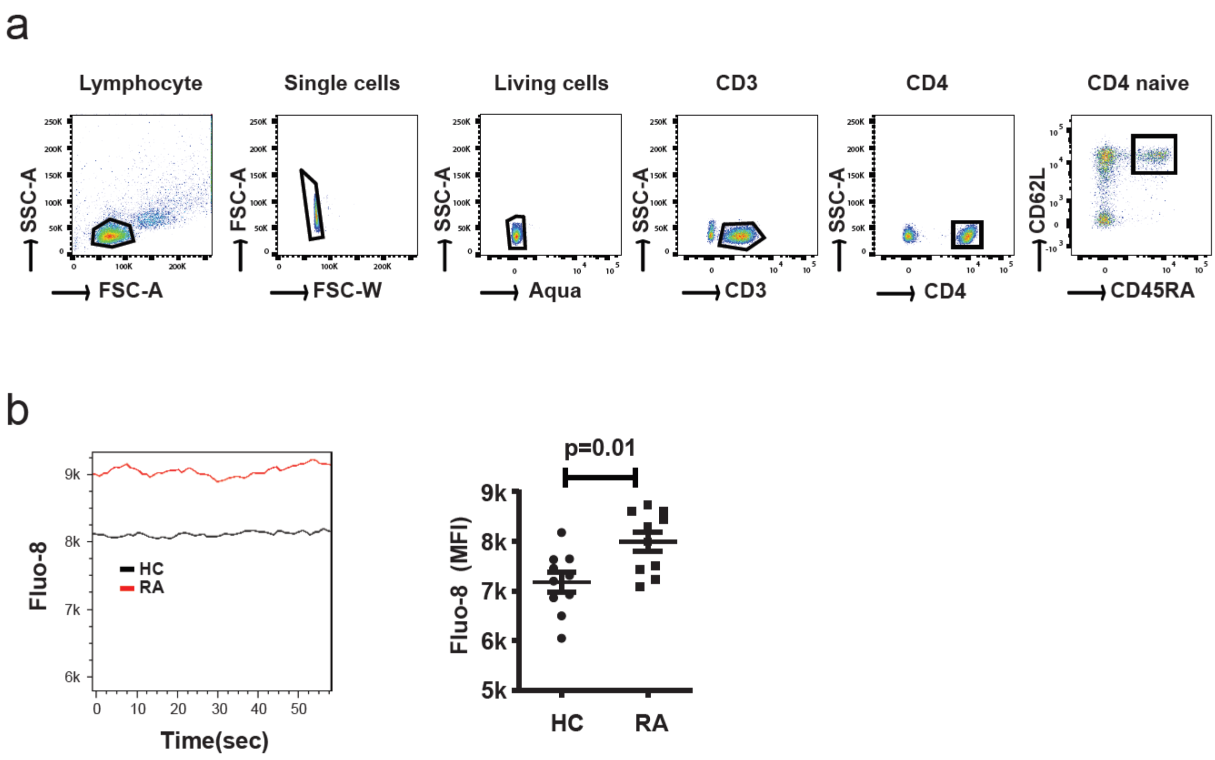

Supplementary Figure 1. Flow cytometric studies of RA T cells. (a) Gating strategies for flow cytometric studies of naïve CD4 T cells from PBMC for pSLP76 and pERK expression corresponding to Fig. 1a, b, d. (b) In initial calcium tracing studies, PBMC cells were labeled with 2 µM Fluo-8 instead of FuraRed. One representative tracing (left) and Fluo-8 fluorescence of naïve $CD3^+CD4^+CD45RA^+CD62L^+$ T cells from 9 HC and 10 RA patients (right). Data were analyzed by unpaired two-tailed student's *t* test and presented as mean± SEM. Gating strategy as in Supplementary Figure 1a. Source data are provided as a Source Data file.

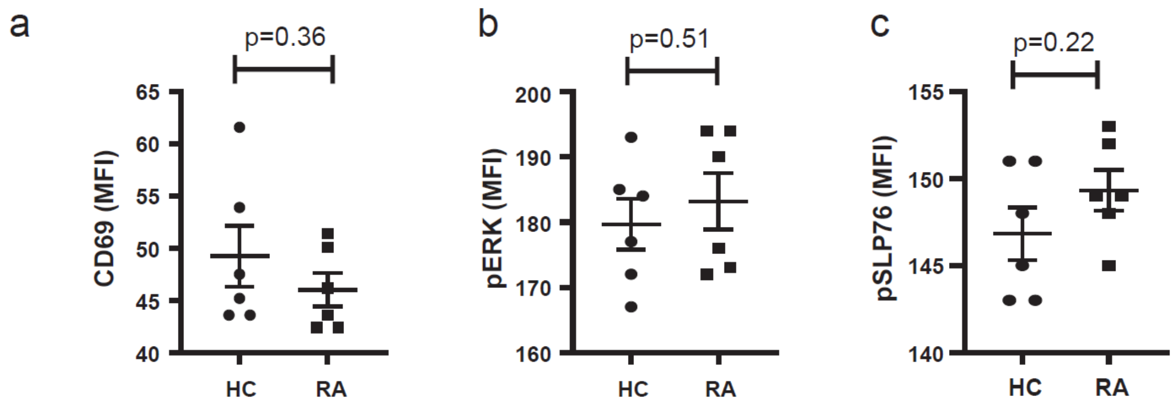

Supplementary Figure 2. Failure to induce activation markers by culture in RA serum. CD4 naïve T cells were cultured with serum from HC (n=6) or RA (n=6) patients for 24 hours. No significant difference in CD69 expression (a) , ERK (b) or SLP76 phosphorylation (c) was observed. Data were analyzed by unpaired two-tailed student's *t* test and presented as mean± SEM (a,b,c). Source data are provided as a Source Data file.

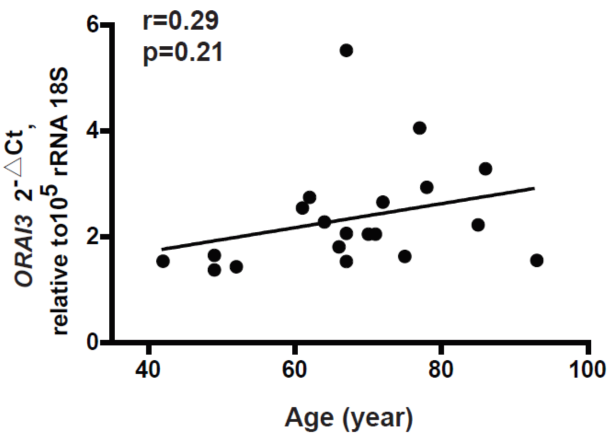

Supplementary Figure 3. Relationship between age and ORAI3 transcript expression in RA patient. Data was analyzed by using two-tailed Pearson’s correlation. Source data are provided as a Source Data file.

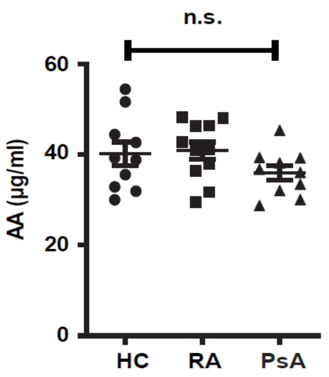

Supplementary Figure 4. Serum concentrations of arachidonic acid. Results are from HC (n=10), RA (n=11) and PsA (n=10) patients. Data were analyzed by unpaired two-tailed student's *t* test and presented as mean ± SEM. Source data are provided as a Source Data file.

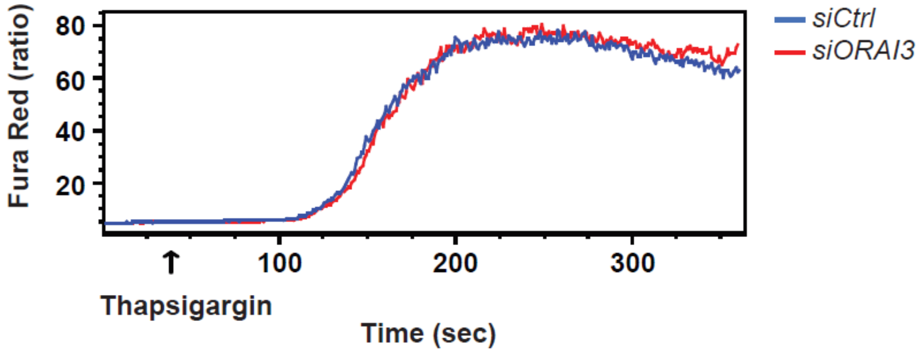

Supplementary Figure 5. Thapsigargin-induced SOCE-dependent calcium flux after *ORAI3* silencing. CD4 naïve T cells were transfected with *ORAI3* or control siRNA. Cells were loaded with Fura-Red. Thapsigargin (2μM)-induced SOCE-dependent calcium influx was monitored.

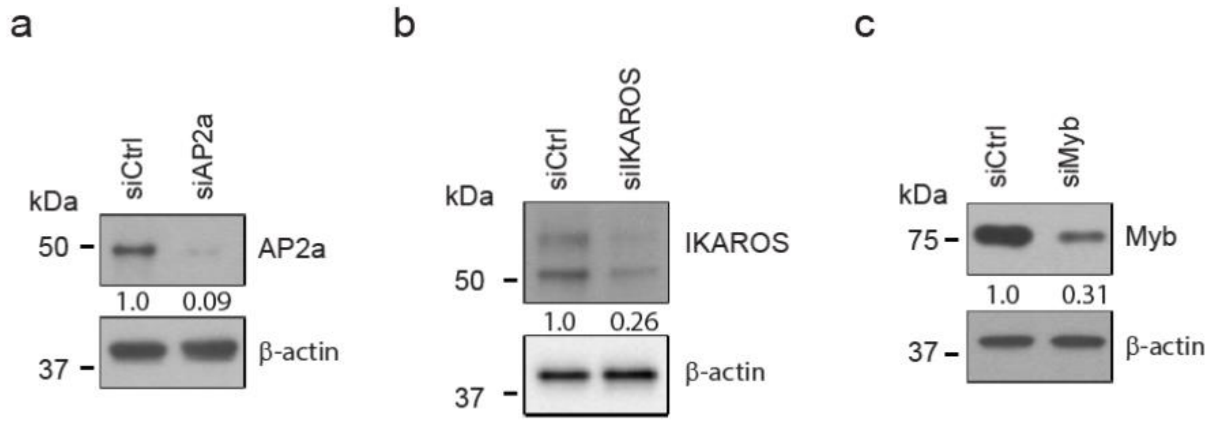

Supplementary Figure 6. Knockdown efficiency of AP-2a, IKAROS and Myb. Western blots corresponding to the experiments in Fig.7b are shown. Two times experiment were repeated independently with similar results. Corresponding uncropped Western blots in Supplementary Figure 10.

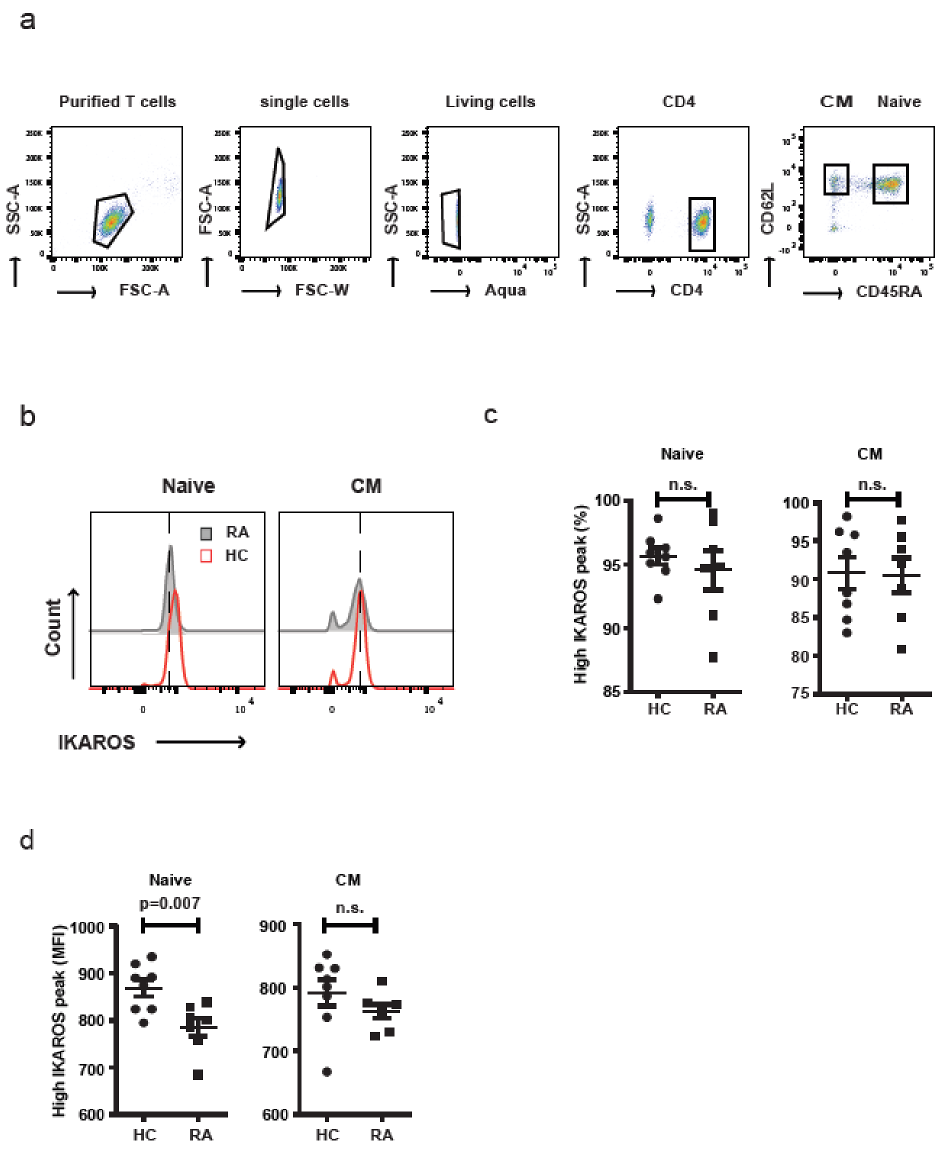

Supplementary Figure 7. Flow cytometric studies of IKAROS expression in naïve and CM CD4 T cells. (a) Gating strategies for flow cytometric studies of naïve and CM CD4 T cells from purified T cells to determine IKAROS expression corresponding to supplementary Fig 7b, c and d. (b) Histograms of IKAROS expression in CD4 T cell subsets of a representative RA patient (top) and a HC (bottom, red). IKAROS expression in CD4 T cell subsets is bimodal with a high and low/negative subpopulation. (c) Frequencies of the IKAROS^{high} population in CD4 subsets from 8 HC and 7 RA patients. (d) IKAROS MFI in the IKAROS^{high} population. Data were analyzed by unpaired two-tailed student's *t* test presented as mean± SEM (c, d). Source data are provided as a Source Data file.

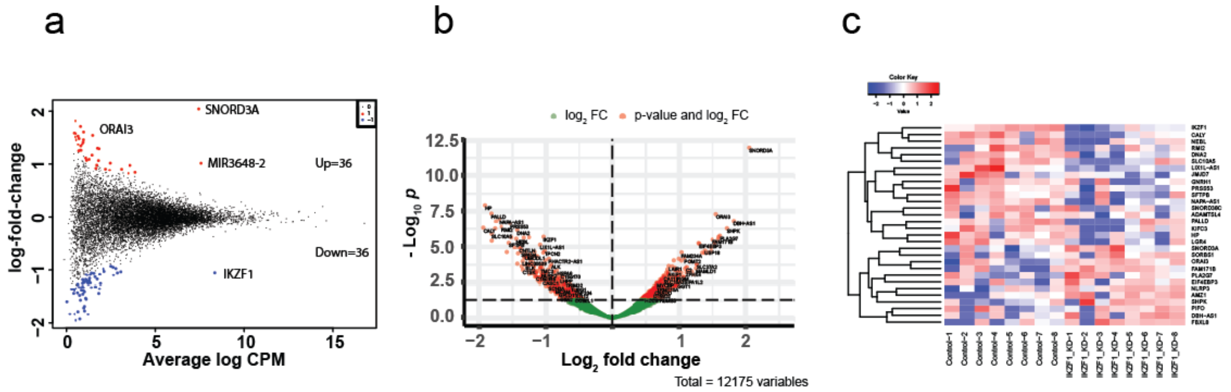

Supplementary Figure 8. Influence of reduced IKAROS expression on the transcriptome.

(a) Purified naïve CD4 T cells from 8 HC were transfected with control or *IKAROS* siRNA and subjected to RNA-seq. Data were analyzed using a negative binomial generalized linear model after conditional quantile normalization. Scatter plot (MA-plot) of $\log_2$ fold differences between control and IKAROS-silenced cells versus the mean of normalized counts. (b) Volcano plot with the top 100 differentially expressed transcripts indicated. (c) Heat plot showing the log CPM z-scores of the top 30 differentially expressed genes.

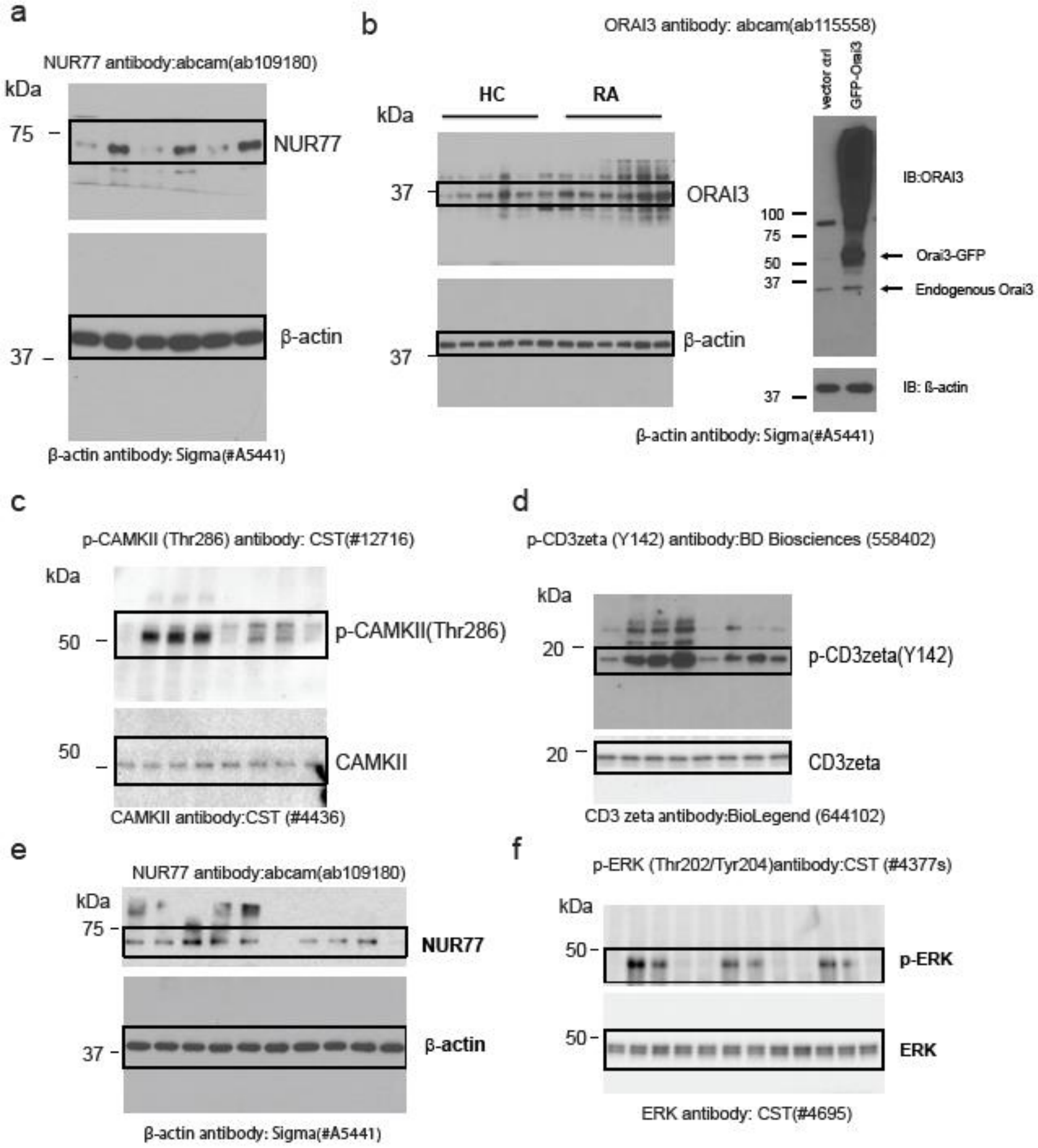

Supplementary Figure 9. Original immunoblots corresponding to Fig. 1e (a); Fig. 2f (b left); Fig. 3b, c &d (c-e), and Fig. 4b (f). Supplementary Figure 2b includes a Western blot of Jurkat cells transfected with control or PCMV- GFP-*ORAI3* plasmid to confirm specificity of the ORAI3-specific antibody (right).

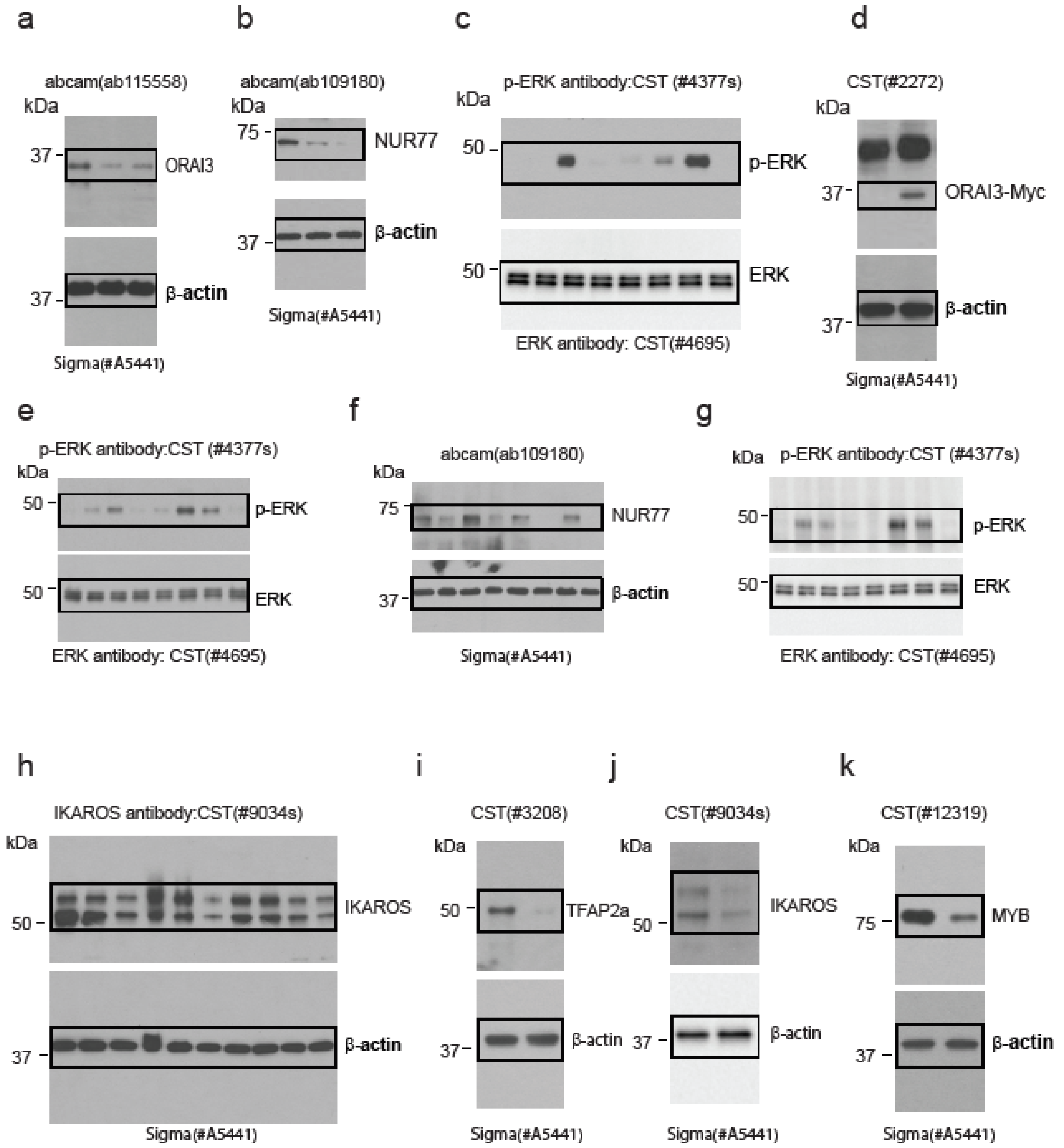

Supplementary Figure 10. Original immunoblot corresponding to Fig. 4c, d, f, g (a-d); Fig. 5b, f (e-f); Fig. 7g (g); Fig. 8c (h); supplementary Figure 6a, b, c (i-k).

Supplementary Table 1.

Demographic and Clinical Characteristics of the Patient Populations

	**Rheumatoid arthritis (RA)**	**Psoriatic Arthritis (PsA)**
**Demographic parameters**		
No. of subjects	97	15
Sex (F/M)	19/78	0/15
Age (mean ± SEM [years])	64.27±1.13	61.2±2.67
**Clinical parameters**		
Disease duration (mean ± SEM [years])	14.57±1.42	11.00±2.34
CDAI (mean ± SEM)	14.48±1.47	17.6±2.61
<2.8 (%)	18	0
2.8 - 10 (%)	26	20
10 – 22 (%)	34	60
>22 (%)	17	20
No DMARD (%)	7	7
Medications (%)		
Corticosteroids	22	7
Methotrexate	43	40
Hydroxychloroquine	45	20
TNFα inhibitor	33	60
Other DMARDs	23	7

Supplementary Table 2. List of primers

Oligo name	Forward (5' – 3')	Reverse (5' – 3')
ITPR1	TTTCCAGAACTGCTTTTGGG	AACCGGGGACCTTAACAATC
ITPR2	CTTGTTTGGCTTGCTTTGCT	CAACCCTCCCAAGAAGTTCA
ITPR3	GAGGCAGTCACGGAACTTCT	GTCAATGGCTTCATCAGCAC
RYR1	AACTGCTTCTGGCTGTCCAT	ACTCAAGGACATTGTGGGCT
RYR2	CCGGAAACAGTATGAAGACCAGCTA	CACACAACGCTGGCAATTCAC
ORAI1	GGTAGTCGTGGTCAGCGTC	CAAGCTTAAAGCCTCCAGCC
ORAI2	ACTGGTACTGCGTCTCCAGC	CTACCTGAGCAGGGCCAA
ORAI3	GGTGGGTACTCGTGGTCACT	GGCCAAGCTCAAAGCTTCC
STIM1	ACACAGGGGCTTGTCAATTC	GTCACAGTGAGAAGGCGACA
STIM2	GCATGGTGGACTCAGTGACA	ACTGGCTCTGCCGCAACT
TRB	CCTTCAACAACAGCATTATTCCAG	CGAGGGAGCACAGGCTGTCTT
TNF	AGATGATCTGACTGCCTGGG	CTGCTGCACTTTGGAGTGAT
TNFSF11	GGTGGATGGCTCATGGTTAGA	CATGTTGGAGATCTTGGCCC
IL6	CTTCGGTCCAGTTGCCTTCT	GTGCCTCTTTGCTGCTTTCA
IL1B	ATCCAGCTACGAATCTCCGA	CCACTTGTTGCTCCATATCC
TBX21	CGGATGTTCCCATTCCTGTC	TTCCACACTGCACCCACTTG
IFNG	ACTAGGCAGCCAACCTAAGCAAGA	CATCAGGGTCACCTGACACATTCA
RORC	TGCCAGAATGACCAGATTGTG	ATGCCACCGTATTTGCCTTC
IL17	CAGGATGCCCAAATTCTGAGG	CAAGGTGAGGTGGATCGGTT
FOXP3	CAGCCATGATCAGCCTCACA	CACTGGGATTTGGGAAGGTG
GATA3	AGGAAGGCATCCAGACCAGA	CCGGGTTAAACGAGCTGTTCT
ACTB	GCACAGAGCCTCGCCTT	GTTGTCGACGACGAGCG
