# Supplementary material for: Arachidonic acid-regulated calcium signaling in T cells from patients with rheumatoid arthritis promotes synovial inflammation
Source: Nat Commun. 2021 Feb 10;12:907. doi: 10.1038/s41467-021-21242-z (PMC7875984; doi:10.1038/s41467-021-21242-z)
Supplement: Supplementary file 3 — Reporting Summary [file 41467_2021_21242_MOESM3_ESM.pdf]

nature research

Corresponding author(s): Jorg J.Goronzy

Last updated by author(s): Nov 25, 2020

Reporting Summary

Nature Research wishes to improve the reproducibility of the work that we publish. This form provides structure for consistency and transparency in reporting. For further information on Nature Research policies, see our Editorial Policies and the Editorial Policy Checklist.

Statistics

For all statistical analyses, confirm that the following items are present in the figure legend, table legend, main text, or Methods section.

n/a	Confirmed	
[ ]	[x]	The exact sample size (*n*) for each experimental group/condition, given as a discrete number and unit of measurement
[ ]	[x]	A statement on whether measurements were taken from distinct samples or whether the same sample was measured repeatedly
[ ]	[x]	The statistical test(s) used AND whether they are one- or two-sided<br>*Only common tests should be described solely by name; describe more complex techniques in the Methods section.*
[x]	[ ]	A description of all covariates tested
[ ]	[x]	A description of any assumptions or corrections, such as tests of normality and adjustment for multiple comparisons
[ ]	[x]	A full description of the statistical parameters including central tendency (e.g. means) or other basic estimates (e.g. regression coefficient) AND variation (e.g. standard deviation) or associated estimates of uncertainty (e.g. confidence intervals)
[ ]	[x]	For null hypothesis testing, the test statistic (e.g. *F*, *t*, *r*) with confidence intervals, effect sizes, degrees of freedom and *P* value noted<br>*Give P values as exact values whenever suitable.*
[x]	[ ]	For Bayesian analysis, information on the choice of priors and Markov chain Monte Carlo settings
[x]	[ ]	For hierarchical and complex designs, identification of the appropriate level for tests and full reporting of outcomes
[x]	[ ]	Estimates of effect sizes (e.g. Cohen's *d*, Pearson's *r*), indicating how they were calculated

Our web collection on statistics for biologists contains articles on many of the points above.

Software and code

Policy information about availability of computer code

Data collection	No software was used for data collection
Data analysis	PRISM 8.4.1(GraphPad), FlowJo_v 10.6.1 or Flowjo_v 9.3.3(Tree Star Inc),). Integrative Genomics Viewer (IGV 2.8.2) software. Promo Transfac 8.3.

For manuscripts utilizing custom algorithms or software that are central to the research but not yet described in published literature, software must be made available to editors and reviewers. We strongly encourage code deposition in a community repository (e.g. GitHub). See the Nature Research guidelines for submitting code & software for further information.

Data

Policy information about availability of data

All manuscripts must include a data availability statement. This statement should provide the following information, where applicable:

- Accession codes, unique identifiers, or web links for publicly available datasets
- A list of figures that have associated raw data
- A description of any restrictions on data availability

All data that support the findings in this study are available from Dr. Goronzy (corresponding author) upon reasonable request. Sequence data are deposited into public data base

Field-specific reporting

Please select the one below that is the best fit for your research. If you are not sure, read the appropriate sections before making your selection.

- [x] Life sciences
- [ ] Behavioural & social sciences
- [ ] Ecological, evolutionary & environmental sciences

For a reference copy of the document with all sections, see nature.com/documents/nr-reporting-summary-flat.pdf

Life sciences study design

All studies must disclose on these points even when the disclosure is negative.

Sample size	The sample size was calculated to ensure 80% power to detect a group difference of 2.0 standard deviations.
Data exclusions	No data were excluded.
Replication	Data were reproduced using various techniques such as qPCR, Western blotting and flow cytometry. All findings reported in this manuscript were reproducible. All experiments were independently replicated with similar results for a minimum of two independent biological replicates.Individual repeats and sample sizes as well as significance levels are described in Figures or Figure legends.
Randomization	N/A for in vitro studies. Experimental mice were randomly assigned to receive control-, ORAI3- or IKAROS-silenced cells
Blinding	Investigator at the time of measurement was blinded to the group assignment.

Reporting for specific materials, systems and methods

We require information from authors about some types of materials, experimental systems and methods used in many studies. Here, indicate whether each material, system or method listed is relevant to your study. If you are not sure if a list item applies to your research, read the appropriate section before selecting a response.

Materials & experimental systems

n/a	Involved in the study	
☐	☒	Antibodies
☐	☒	Eukaryotic cell lines
☒	☐	Palaeontology and archaeology
☐	☒	Animals and other organisms
☐	☒	Human research participants
☐	☒	Clinical data
☒	☐	Dual use research of concern

Methods

n/a	Involved in the study	
☒	☐	ChIP-seq
☐	☒	Flow cytometry
☒	☐	MRI-based neuroimaging

Antibodies

Antibodies used

Dilutions Used: FACS: Antibodies for flow cytometry were used at a concentration of 1:100 unless recommended otherwise by the manufacturer. Western blots: Antibodies used in immunoblotting were used at a concentration of 1:1000 (primary antibody) or 1:5,000 (anti-mouse HRP secondary antibody) or 1:10,000 (anti-rabbit HRP secondary antibody) unless recommended otherwise by the manufacturer. Primary antibodies for Immunobiochemistry were used at a concentration of 1:50 -1:100. Secondary antibodies were used at a concentration of 1:500(DyLight® 594 Anti-Mouse IgG or DyLight® 488 Anti-Rabbit IgG).
Antibodies used in chromatin immunoprecipitation studies were used at 5 ug/sample.
Antibody List:
Western blot/ChIP:
Phospho-CaMKII (Thr286) (D21E4) Rabbit mAb #12716, Cell Signaling
Phospho-p44/42 MAPK (Erk1/2) (Thr202/Tyr204) (197G2) Rabbit mAb #4377s, Cell Signaling
CaMKII (pan) (D11A10) Rabbit mAb #4436, Cell Signaling
p44/42 MAPK (Erk1/2) Antibody #4695 Cell Signaling
c-Myb (D2R4Y) Rabbit mAb #12319 Cell Signaling
AP-2α Antibody #3208 Cell Signaling
Myc Antibody #2272 Cell signaling
Ikaros (D10E5) Rabbit mAb #9034s Cell Signaling
Monoclonal Anti-β-Actin antibody (clone AC-15) #A5441 Sigma
Anti-Orai3 antibody ab115558 Abcam
Recombinant Anti-NUR77 antibody (EPR3209) ab109180 Abcam
Purified Mouse anti-CD247 (pY142) (CD3 (Clone K25-407.69) 558402 BD Biosciences
Purified anti-CD247 (CD3ζ) Antibody(6B10.2) 644102 BioLegend
mouse anti-rabbit IgG-HRP sc-2357 Santa Cruz

	Anti-mouse IgG, HRP-linked Antibody #7076 Cell signaling<br>Flow cytometry:<br>V450 Mouse Anti-Human CD4 (Clone RPA-T4) 560345 BD Biosciences<br>PE-Cy™7 Mouse Anti-Human CD8 (Clone RPA-T8) 557746 BD Biosciences<br>Alexa Fluor® 700 Mouse Anti-Human CD45RA (Clone HI100) 560673 BD Biosciences<br>PerCP-Cy™5.5 Mouse Anti-Human CD69 (Clone FN50) 560738 BD Biosciences<br>Alexa Fluor® 647 Mouse Anti-ERK1/2 (pT202/pY204) (Clone 20A) 561992 BD Biosciences<br>Alexa Fluor® 647 Mouse anti-SLP-76 (pY128) (Clone J141-668.36.58) 558438 BD Biosciences<br>PE anti-human CD62L Antibody (Clone DREG-56) 304806 Biolegend<br>Alexa Fluor® 647 anti-human Ikaros Antibody (Clone 16B5C71) 368404 Biolegend<br>Alexa Fluor® 488 anti-human CD3 Antibody (Clone UCHT1) 30045 Biolegend<br>In vitro T cell activation:<br>Ultra-LEAF™ Purified anti-human CD3 Antibody (OKT3) 317347 Biolegend<br>Ultra-LEAF™ Purified anti-human CD28 Antibody (CD28.2) 302943 Biolegend<br>Immunohistochemistry:<br>Monoclonal Mouse Anti-Human CD3 (Clone F7.2.38) M725429-2 Agilent Dako<br>Anti-Interferon gamma antibody ab25101 Abcam<br>VectaFluor™ Excel Amplified DyLight® 594 Anti-Mouse IgG Kit DK-2594 Vector Laboratories<br>VectaFluor™ Excel Amplified DyLight® 488 Anti-Rabbit IgG Kit DK-1488 Vector Laboratories
Validation	All antibodies used for human samples have been validated by the respective company. If not, we run validation assays.Further details in validation can be found in Biolegend Reproducibility and Validation webpage (https://www.biolegend.com/en-us/reproducibility),Cell Signaling Technology (https://www.cellsignal.com/contents/_/cstantibody-validation-principles/ourapproach-validation-principles) , Abcam (https://www.abcam.com/primary-antibodies/a-guide-to-antibody-validation) and Santa Cruz Technologies (https://www.labome.com/ method/Santa-Cruz-Antibodies.html)

Eukaryotic cell lines

Policy information about cell lines

Cell line source(s)	Jurkat T cell line and HEK293T cells were purchased from ATCC
Authentication	None of the cell lines was authenticated
Mycoplasma contamination	Cell lines were not tested for mycoplasma contamination
Commonly misidentified lines (See ICLAC register)	None

Animals and other organisms

Policy information about studies involving animals; ARRIVE guidelines recommended for reporting animal research

Laboratory animals	NOD.Cg-PrkdcscidIl2rgtm1Wjl/SzJ (NSG) mice (Jackson Laboratory, Bar Harbor, ME) were kept in pathogen-free facilities and used at the age of 8-12 weeks. Husbandry is performed in accordance with the Guide for the Care and Use of Laboratory Animals and the Public Health Service Policy on Humane Care and Use of Laboratory Animals. Room conditions included a temperature of 23 °C ± 2 °C, relative humidity of 30% to 40%, and a 12:12-h light:dark cycle.
Wild animals	None
Field-collected samples	None
Ethics oversight	All animal experiments were approved by the Palo Alto Veterans Administration Healthcare System Animal Care and Use Committee

Note that full information on the approval of the study protocol must also be provided in the manuscript.

Human research participants

Policy information about studies involving human research participants

Population characteristics	Patients with anti-CCP-positive rheumatoid arthritis and patients with psoriatic arthritis. Healthy controls recruited from community volunteers. Demographics as shown in the manuscript. see Supplemental Table 1.
Recruitment	Patients were recruited from the Palo Alto VA Rheumatology Clinic by treating physician.
Ethics oversight	Stanford University Institutional Review Board.

Note that full information on the approval of the study protocol must also be provided in the manuscript.

Clinical data

Policy information about clinical studies

All manuscripts should comply with the ICMJE guidelines for publication of clinical research and a completed CONSORT checklist must be included with all submissions.

Clinical trial registration	N/A
Study protocol	N/A
Data collection	Cross-sectional study. Clinical data were abstracted from data obtained at the time of the clinical visit.
Outcomes	N/A

Flow Cytometry

Plots

Confirm that:

- [x] The axis labels state the marker and fluorochrome used (e.g. CD4-FITC).
- [x] The axis scales are clearly visible. Include numbers along axes only for bottom left plot of group (a 'group' is an analysis of identical markers).
- [x] All plots are contour plots with outliers or pseudocolor plots.
- [x] A numerical value for number of cells or percentage (with statistics) is provided.

Methodology

Sample preparation	Peripheral blood mononuclear cells were isolated by gradient centrifugation. T cell subpopulations were isolated from PBMC using enrichment kits from STEMCELL Technologies as described in the Methods section.
Instrument	LSR II or Fortessa cytometer (BD Biosciences).
Software	FlowJo
Cell population abundance	Isolated cell populations were >95% pure.
Gating strategy	Naive CD4 T cells were gated as CD4+CD45RA+CD62L+, central memory CD4 T cells were gated as CD4+CD45RA-CD62L+. See supplementary Figure 1a, 7a.

- [x] Tick this box to confirm that a figure exemplifying the gating strategy is provided in the Supplementary Information.
